# Anaemia among children in a drought affected community in south-central Ethiopia

**DOI:** 10.1371/journal.pone.0170898

**Published:** 2017-03-14

**Authors:** Taye Gari, Eskindir Loha, Wakgari Deressa, Tarekegn Solomon, Hanibale Atsbeha, Meselech Assegid, Alemayehu Hailu, Bernt Lindtjørn

**Affiliations:** 1 School of Public and Environmental Health, College of Medicine and Health Sciences, Hawassa University, Hawassa, Ethiopia; 2 Centre for International Health, University of Bergen, Bergen, Norway; 3 Department of Preventive Medicine, School of Public Health, College of Health Sciences, Addis Ababa University, Addis Ababa, Ethiopia; 4 School of Medicine, College of Medicine and Health Sciences, Hawassa University, Hawassa, Ethiopia; 5 Department of Reproductive Health and Health Service Management, School of Public Health, College of Health Sciences, Addis Ababa University, Addis Ababa, Ethiopia; TNO, NETHERLANDS

## Abstract

**Introduction:**

As part of a field trial (PACTR201411000882128) to provide evidence on the combined use of long-lasting insecticidal nets and indoor residual spray for malaria prevention, we measured haemoglobin values among children aged 6 to 59 months. The aim of this study was to estimate the prevalence of anaemia, and to determine the risk factors of anaemia and change in haemoglobin value in Adami Tullu district in south-central Ethiopia.

**Methods:**

Repeated cross-sectional surveys among 2984 children in 2014 and 3128 children in 2015; and a cohort study (malaria as exposure and anaemia as outcome variable) were conducted. The study area faced severe drought and food shortages in 2015. Anaemia was diagnosed using HemoCue Hb 301, and children with haemoglobin <11 g/dl were classified as anaemic. Multilevel and Cox regression models were applied to assess predictors of anaemia.

**Results:**

The prevalence of anaemia was 28.2% [95% Confidence Interval (CI), 26.6–29.8] in 2014 and increased to 36.8% (95% CI, 35.1–38.5) in 2015 (P<0.001). The incidence of anaemia was 30; (95% CI, 28–32) cases per 100 children years of observation. The risk of anaemia was high (adjusted Hazard Ratio = 10) among children with malaria. Children from poor families [Adjusted Odds Ratio (AOR); 1.3; 95% CI, 1.1–1.6)], stunted children (AOR 1.5; 95% CI; 1.2–1.8), and children aged less than 36 months (AOR; 2.0; 95% CI, 1.6–2.4) were at risk of anaemia compared to their counterparts. There was no significant difference in risk of anaemia among the trial arms.

**Conclusions:**

Young age, stunting, malaria and poverty were the main predictors of anaemia. An increase in the prevalence of anaemia was observed over a year, despite malaria prevention effort, which could be related to the drought and food shortage. Therefore, conducting trials in settings prone to drought and famine may bring unexpected challenges.

## Introduction

Anaemia is a common childhood health problem in Africa, and its prevalence among children under the age of five years is estimated at 62%, which is above the cut off points (40%) of the World Health Organization (WHO) classification of anaemia as a severe public health problem [1]. Anaemia has a serious effect on child health, and could result in impaired cognitive function, poor school performance, poor growth and development, and threatens the life of children [2–4]. The risk factors of anaemia are multiple, and vary across geographical areas. Iron deficiency anaemia is the leading (50%) cause of childhood anaemia in developing countries [5, 6]. In such countries, increased risks for childhood anaemia are protein energy malnutrition [7] and infections such as malaria, diarrhoea and intestinal helminths [7–12]. In addition, poverty [9, 13], illiteracy [14], and poor hygiene and sanitation [15] are among the contributing factors for the occurrence of anaemia.

In Ethiopia, the estimated national prevalence of anaemia among children aged 6 to 59 months was about 44% in 2011, and classified as a severe public health problem [16, 17]. To control childhood anaemia, the Ethiopian Ministry of Health (MOH) has been implementing anaemia prevention integrated into the routine child health services and community based nutrition intervention programmes. In addition, the MOH has carried out periodic de-worming campaigns of children since 2004 [18]. However, despite the concerted efforts to control childhood anaemia over the last two decades, anaemia still remains a major childhood health problem in the country [16].

Repeated community-based cross-sectional measurement on the same population has been used to study the effects of anaemia control interventions [19–21]. Unfortunately, such repeated measurements of anaemia prevalence have not been done in Ethiopia [3, 7, 22]. As part of a field trial to provide evidence on the combined use of long-lasting insecticidal nets (LLINs) and indoor residual spraying (IRS) for malaria prevention, we measured haemoglobin values among children aged 6 to 59 months [23]. The objectives of this study were to estimate the prevalence of anaemia, and to assess the risk factors of anaemia, and to measure changes in the haemoglobin concentration among children followed over a period of one year.

## Methods

### Study setting and profile

**Fig 1:**
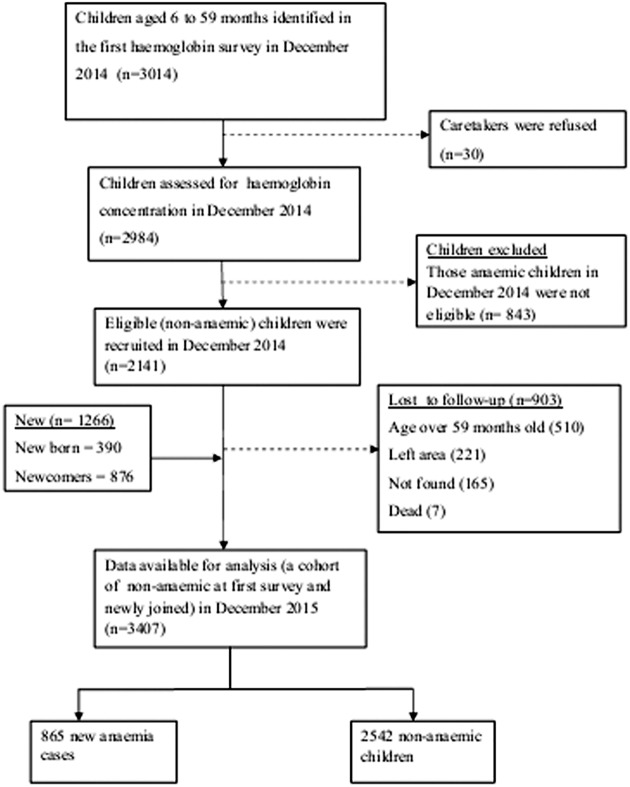
Study profile in Adami Tullu district in south-central Ethiopia, 2014 and 2015.

**Fig 2:**
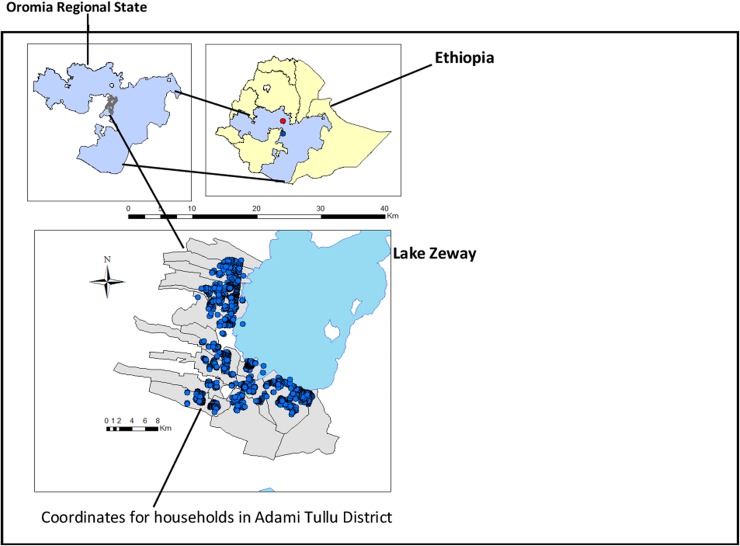
Map of Adami Tullu district in Oromia Regional State in south-central Ethiopia. Re-print under a CC BY license, with permission from Deressa et al Trials (2016).

The study profile was presented in Fig 1, and this study was conducted in Adami Tullu district in the Oromia Regional State in south-central Ethiopia. The district is situated in the Great Rift Valley and has 48 "*Kebeles"* (lowest government administrative unit). Each *kebele* is further divided into villages or *gares*, and about 1000 to 5000 people live in a *kebele* [24]. Lake Zeway, a potential breeding site for malaria vectors, borders most of the study *kebeles* (Fig 2). In 2014, the population of the district was projected to be about 173,000 people, and children under the age of five years accounted for 12% of the population [24]. The livelihood of the households is subsistence agriculture and cattle rearing. Most of the families are dependent on rain-fed agriculture.

Like many districts in Ethiopia, our study area was affected by repeated droughts and food shortage during the past decades [25]. The population also faced severe drought triggered by El Nino in 2015 and early 2016 [26]. The annual rainfall of the district was 673 mm in 2011, 909 mm in 2012, 745 mm in 2013, 673 mm in 2014 (only 8 months’ data available) and decreased to 471 mm in 2015. The average maximum temperature was 27 C in 2014, and 29 C in 2015 [27]. The communities were receiving food aids, and a non-governmental organization was also screening and treating children with acute malnutrition. Malaria is a common health problem in the district [28]. There is one health post staffed by two health extension workers in each *kebele*. Malaria prevention, diagnosis and treatment with anti-malaria drugs, and periodic de-worming of children under five years to control anaemia are among the services given in the health post.

### Study design and participants

This study is a part of a cluster randomized controlled trial to measure the combined use of LLINs and IRS over LLINs or IRS alone to prevent malaria [23]. In the anaemia study, repeated cross-sectional surveys, and a cohort study were conducted. In the cross-sectional study, we conducted surveys to estimate the prevalence of anaemia, and anthropometry measurement in December 2014 (n = 2984 children), followed by the second anaemia and anthropometry survey in December 2015 (n = 3128 children). During the two surveys, we included all children in the age group of 6 to 59 months old in all villages that were enrolled to malaria prevention trials.

Two cohort groups were identified and enrolled in December 2014. In the first cohort, non-anaemic children were recruited in December 2014 and were followed for a year. The main exposure variable was malaria, and the outcome variable was anaemia. A haemoglobin measurement survey was conducted to diagnose anaemia at the end of a year follow-up. This is a dynamic cohort where older children left the study, while children born during the study period and newcomers joined the cohort. The dynamic cohort included 2141 non-anaemic children diagnosed in December 2014, and 1266 children added during the follow-up period, making a total of 3407 children (Fig 1).

The second (closed) cohort was a study in which only children participating in both surveys were included. Among the 1851 children participating in both surveys, 1244 children were non-anaemic in December 2014, and these children were also participated in the second survey in December 2015. This was a closed cohort, where children who participated in both surveys were included, and children who left the area, children aged over 59 months old and newcomers were excluded.

The study design and the sample size estimation for the malaria prevention trial was described by Deressa et al [23], and included all children aged 6 to 59 months old from 6070 households in 176 villages from 13 *kebeles* living within 5 km from Lake Zeway in the Adami Tullu district. The assumption of sample size determination for haemoglobin concentration was that the combination of IRS and LLINs could increase the mean haemoglobin by 0.5 g/dl compared to either IRS or LLINs alone. A study conducted in south-west Ethiopia showed a mean (standard deviation) haemoglobin of 11.5 (1.66) g/dl [7]. Assuming 90% power, 5% significance level, and a design effect of two, the sample size estimated to be 464 children per arm of the trial, resulting in a total of 1856 children for the whole study. Although the estimated sample size sufficiently addresses the research question in this study, we included and followed-up all (n = 2984) children aged 6 to 59 months participating in all arms of the main trail.

### Data collection

Interviewer administered, pre-tested structured questionnaires were used to collect data on socio-demographic and economic variables. Data were collected by diploma graduates, whereas malaria diagnosis and treatment, anthropometric and haemoglobin measurements were conducted by nurses. The nurses were given skill-based training on how to take anthropometric measurements, finger-pricks, blood samples, malaria diagnosis using rapid diagnostic tests (RDTs), haemoglobin measurement, and treatment of malaria cases.

### Blood tests for haemoglobin and malaria

Blood samples were obtained from finger-pricks, and haemoglobin concentration was measured using HemoCue Hb 301 (HemoCue AB, Angelholm, Sweden). Based on the manufacturer's instruction, micro-cuvette was filled in with 10μL drops of capillary blood, placed in the HemoCue analyser and the result read after 10 seconds. The WHO criteria were used to define and classify anaemia in children aged 6 to 59 months [29]. Anaemia was defined as haemoglobin <11 g/dl, and classified into mild anaemia if the haemoglobin concentration was in the range of 10–10.9 g/dl, moderate anaemia if haemoglobin concentration was 7–9.9 g/dl and severe if the haemoglobin concentration was <7 g/dl.

Febrile patients were identified through weekly home visit and referred to the nearest health posts for malaria diagnosis and treatment. Capillary blood samples were collected from children presented to health post with clinical sign of malaria for RDTs to diagnose malaria, which was performed using CareStart ^TM^ produced by Premier Medical Corporation Limited in India. Patients with malaria were treated according to the national malaria treatment guideline [30].

### Anthropometric measurements

The data collection teams were trained for two days, and the measurement techniques were standardized before each survey. Each observer measured the weight and height or length of 10 children twice. The inter and intra technical errors of measurements (TEM) were within suggested cut-off points for acceptability of measurements [31]. The intra TEM was 0.08 Kg for weight and 0.14 cm for height, whereas the inter TEM was 0.1 Kg for weight and 0.2 cm for height. The weight was taken using Salter scale, which was calibrated daily and adjusted to zero. A standard wooden board was used to measure the height of children older than 2 years, and recumbent length for those children less than 2 years of age. As most parents did not know their child's date of birth, the parents or caregivers were probed using a local event calendar to obtain an approximate age [32]. The WHO 2006 multi-centre growth reference study [33] was used to calculate nutritional indices such as weight-for- height (WHZ), height- for-age (HAZ) and weight-for-age (WAZ) by using Emergency Nutrition Assessment for SMART software 2011 [34]. Children were classified as wasted (WHZ <-2 Z-scores), stunted (HAZ <-2 Z-scores) and underweight (WAZ <-2 Z-scores).

### Statistical analysis

Data were entered into SPSS version 21 (SPSS Inc., Chicago, USA), and also analysed using STATA version 14 (StataCorp, College Station, TX, USA). Descriptive statistics, frequency count, percentage, mean and standard deviation were computed. Principal component analysis (PCA) was used to construct a wealth index [35] from 14 household assets related variables such as electricity, television, radio, mobile telephone, table, chair, bed, separate kitchen from living house, types of roof and walls and ownership of a bicycle, animal cart, animal and land. These variables were dichotomized and coded "1" if the household owned the asset or "0” if not. The Kaiser-Meyer-Olkin (KMO) measure of sample adequacy was 0.79. The first principal component represented 23.6% of the variance in the sample with an Eigen value of 3.3 and categorized into three relative measures of socioeconomic classes (poor, middle and Rich).

To measure risk factors of anaemia among children participating in the cross-sectional surveys, a multilevel model was used to account for clustering within a group at different level [36]. In this study, predictors of anaemia were clustered at two levels; individual child (first level) was nested to households or families with the assumption of differences in risk of anaemia between families but similarity among children within a family. Based on this assumption, the presence of clustering was checked before fitting multilevel model in the following steps: First, a null single level (standard) regression model, and then a null multilevel model with the random household effect were fitted. The calculated likelihood ratio test statistics showed strong evidence of household effect on anaemia status of the children (P<0.001). Hence, to account for the clustering, a multilevel model was fitted to estimate crude odds ratios (OR) and adjusted odds ratios (AOR) with 95% CI). We did not adjust for altitude, because the households were located between 1613 and 1758 above sea level, which was assumed to be relatively homogeneous.

We calculated anaemia incidence for the closed cohort, considering the number of children that were diagnosed with anaemia in 2015, among those without these conditions in 2014 survey. Whereas, the incidence rate for the dynamic cohort was calculated considering children that were anaemic in 2015 among those non-anaemic children. These non-anaemic children were those identified in December 2014 survey and those children newly joined the cohort during the follow-up period. Cox regression model was fitted to measure the hazard ratio (HR), malaria was entered as a time varying covariate for anaemia, and socio-demographic factors such as sex, wealth status and educational status were entered as an independent predictor variables for anaemia. Bivariate analysis was computed for all variables. All variables with P< 0.25 were fitted to the model and those with P <0.05 were maintained and reported.

### Ethical issues

Ethical clearance was obtained from the Institutional Review Board of the College of Health Sciences at Addis Ababa University, the National Ethics Committee of the Ministry of Science and Technology in Ethiopia (ref: 3.10/446/06), and from the Regional Committee for Medical and Health Research Ethics, Western Norway (ref: 2013/986/REK Vest). Written permission was obtained from the Oromia Regional Health Bureau (ORHB), East Shewa Zone Health Department (ESZHD) and Adami Tullu District Health Office. Consultative meeting and discussion was made before the trial implementation with representatives from Adami Tullu District Administration, ESZHD and ORHB. In addition, *Kebele* and village leaders and community elders were also sensitized through meeting before the study. During the meeting and sensitization the objectives, randomization, implementation process and expected outcome of the trial were discussed. As the majority of the study population cannot read and write, we had a challenge to get written consent. Therefore, verbal informed consent was obtained from the head of households, or members of the households older than 18 years in the absence of the head of the households at the beginning of our cluster randomized controlled trial [23]. In addition, the verbal consent was obtained from the parents or caretakers before collecting the blood samples from the children. We used standard information sheet to explain the purpose of the study, and the participants were informed that participation was voluntary and that they had the right to withdraw any time during the study. They were assured that refusal to participate in the study would not affect their health service utilization at the health posts.This information was read to them using the information sheet in their own language, and their consent was recorded using check (√) mark. We also strictly supervised the data collectors, whether they are following the information sheets or not. Later, this document was stored at our field research station. Children with positive RDT confirmed malaria, and those with uncomplicated severe acute malnutrition were treated in the health post according to the national malaria and malnutrition treatment guidelines [30, 37]. Those children with anaemia were referred to the nearest health centre.

## Results

### Characteristics of study children

A total of 2984 children in the 2014 survey, and 3128 children in the 2015 survey were included. The mean (SD) age was 33.6 (14.6) months in 2014, and 35.2 (15) months in 2015. In the 2014, the proportion of boys was 51.3%, and 56.6% of the head of households were illiterate (Table 1).

**Table 1:** Demographic and anthropometric measurements of children, and household characteristics in Adami Tullu district in south-central Ethiopia, 2014 and 2015.

Variables		2014 (n = 2984)	2015 (n = 3128)
		Number (%)	Number (%)
Sex	Boys	1532 (51.3)	1586 (50.7)
	Girls	1452 (48.7)	1542 (49.3)
Age in months	6–35	1499 (50.2)	1531 (49.0)
	36–59	1485 (49.8)	1597 (51.0)
	Mean age (95% CI)	33.6 (33.0–34.1)	35.2 (34.6–35.7)
Wealth status	Poor	955 (32.0)	966 (32.1)
	Middle	1016 (34.1)	1040 (34.6)
	Rich	1012 (33.9)	1004 (33.3)
Household head education	Illiterate	1689 (56.6)	1856 (59.4)
	Elementary	971 (32.5)	926 (29.6)
	High school and above	324 (10.9)	343 (11.0)
Weight-for height	<-2 Z-score	215 (7.3)	125 (4.1)
	≥-2 Z-score	2737 (92.7)	2953 (95.9)
Height- for- age	<-2 Z-score	1323 (44.8)	1562 (50.7)
	≥ -2 Z-score	1629 (55.2)	1516 (49.3)
Weight-for-age	<-2 Z-score	552 (18.5%)	481 (15.4%)
	≥ -2 Z-score	2432 (81.5%)	2647 (84.6%

CI: Confidence Interval

### Prevalence of malnutrition

The prevalence of stunting (height-for-age <-2 Z-score) was 44.8% in 2014, and increased to 50.7% in 2015 (P <0.001). The prevalence of underweight was 18.5% in 2014, and 15.4% in 2015 (P<0.001). Whereas, the prevalence of wasting was 7.3% in 2014, and 4.1% in 2015 (P<0.001) (Table 1).

### Prevalence of anaemia

The prevalence of anaemia was 28.2% (95% CI, 26.6–29.8) in 2014, and increased to 36.8% (95% CI; 35.1–38.5) in 2015 (P< 0.001). The mean haemoglobin value decreased from 11.6 g/dl in 2014 to 11.2 g/dl in 2015 (P<0.001). The decrease in haemoglobin value (P<0.05) was observed for all factors included in this study (Table 2).

**Table 2:** The mean haemoglobin values, and prevalence of anaemia among children in Adami Tullu district in south-central Ethiopia, 2014 and 2015.

Variables		2014 (n = 2984)		2015 (n = 3128)		P-value†
		HB	Anaemia prevalence	HB	Anaemia prevalence	
		Mean (SD)	Number (%)	Mean (SD)	Number (%)	
Sex	Boys	11.6 (1.7)	441 (28.8)	11.2 (1.7)	592 (37.4)	<0.001
	Girls	11.6 (1.7)	400 (27.5)	11.2 (1.7)	559 (36.2)	<0.001
Wealth status	Poor	11.5 (1.8)	300 (31.4)	11.1 (1.7)	371 (38.4)	<0.001
	Middle	11.6 (1.6)	281 (27.7)	11.2 (1.7)	405 (38.9)	<0.001
	Rich	11.7 (1.7)	259 (25.6)	11.4 (1.7)	333 (33.2)	<0.001
Age in months	6–35	11.2 (1.6)	527 (35.2)	10.7 (1.6)	705 (46.0)	<0.001
	36–59	11.9 (1.7)	314 (21.1)	11.4 (1.7)	446 (28.0)	<0.001
Household's head education	Illiterate	11.5 (1.7)	484 (28.8)	11.2 (1.7)	720 (38.9)	<0.001
	Elementary	11.6 (1.6)	260 (27.4)	11.4 (1.7)	317 (33.7)	0.008
	Secondary and above	11.8 (1.6)	87 (26.9)	11.3 (1.5)	114 (33.2)	<0.001
Weight-for-age	<-2 Z-score	11.2 (2.0)	200 (36.2)	10.9 (1.8)	204 (42.4)	<0.001
	≥-2 Z-score	11.6 (1.6)	641 (26.4)	11.3 (1.7)	947 (35.8)	<0.001
Height-for-age	<-2 Z-score	11.3 (1.8)	468 (34.5)	11.0 (1.7)	642 (41.8)	<0.001
	≥-2 Z-score	11.9(1.5)	372 (22.9)	11.5 (1.7)	509 (32.0)	<0.001
Weight-for-height	<-2 Z-score	11.5 (2.0)	62 (27.1)	11.4 (1.7)	37 (29.6)	0.320
	≥ -2z-score	11.6 (1.6)	777 (28.2)	11.2 (1.7)	1114 (37.1)	<0.001
Intervention arm	IRS + LLINs	11.7 (1.5)	199 (26.8)	11.1 (1.7)	310 (38.1)	<0.001
	LLINs alone	11.5 (1.7)	220 (28.6)	11.2 (1.8)	282 (35.0)	<0.001
	IRS alone	11.5 (1.7)	199 (29.1)	11.2 (1.7)	272 (38.5)	<0.001
	Routine	11.5 (1.7)	223 (28.3)	11.4 (1.7)	287 (35.8)	>0.05

HB: Haemoglobin concentration.

†: t-test was used to compare the mean HB values of the two surveys.

SD: Standard Deviation.

**Fig 3:**
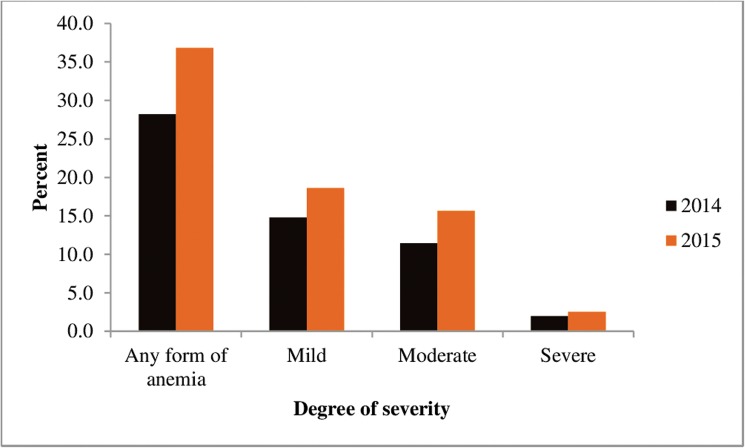
Severity of anaemia among children in Adami Tullu district in south-central Ethiopia, 2014 and 2015.

As shown in Fig 3, the prevalence of all types of anaemia, increased over a year period. Mild anaemia prevalence increased from 14.8% in 2014 to 18.6% in 2015 (P<0.001), and moderate anaemia prevalence increased from 11.4% in 2014 to 15.7% in 2015 (P<0.001).

### Risk factors associated with anaemia

Bivariate multilevel mixed effect model analysis showed that poor socioeconomic status (OR = 1.4; 95% CI, 1.1–1.7), stunting (OR = 1.9; 95% CI, 1.6–2.2) and age less than 36 months old (OR = 2; 95% CI, 1.6–2.4) were risk factors of anaemia in 2014. Likewise, in 2015, educational status of head of household (OR = 1.3; 95% CI, 1.0–1.7) was a risk factor of anaemia in addition to the risk factors identified in 2014.

The multivariate multilevel mixed effect model analysis showed that children living in a poor family (AOR = 1.3; 95% CI, 1.1–1.6), children who were stunted (AOR = 1.5; 95% CI, 1.4–1.9) and children aged less than 36 months (AOR = 2.0; 95% CI, 1.6–2.4) were at risk of anaemia in 2014. Similarly, in 2015, children living in poor families (AOR = 1.3; 95% CI, 1.03–1.6), children who were stunted (AOR = 1.6; 95% CI, 1.3–1.9), children aged less than 36 months (AOR = 2.4;95% CI, 2.0–2.9) and where the head of the household was illiterate (AOR = 1.5; 95% CI, 1.1–2.0) were at increased risk of anaemia. However, wasting was not a risk factor of anaemia during the two surveys, and no statistically significant difference in anaemia was observed among malaria prevention trial arms (Table 3).

**Table 3:** Multilevel logistic regression for predictors of anaemia among children in Adami Tullu district in south-central Ethiopia, 2014 and 2015.

Variables		2014 (n = 2984)			2015 (n = 3128)		
		Anaemic	Unadjusted † OR (95% CI)	Adjusted OR £ (95% CI)	Anaemic	Unadjusted † OR (95% CI)	Adjusted OR £ (95% CI)
		Cases N (%)			Cases N (%)		
**Sex**	Boys	440 (28.7)	1.1 (0.6–0.9)	NA	593 (37.4)	1.1 (0.9–1.2)	NA
	Girls	401 (27.6)	1.0	NA	558 (36.2)	1.0	NA
**Age in months**	6–35	526 (62.5)	2.2 (1.8–2.6)*	2.0 (1.7–2.4)*	705 (46.0)	2.4 (2.0–2.9) *	2.5 (2.1–3.0) *
	36–59	315 (37.5)	1.0	1.0	446 (28.0)	1.0	1.0
**Height-for-age**	<-2 Z-scores	468 (34.5)	1.9 (1.6–2.2)*	1.6 (1.4–2.0)*	642 (41.8)	1.6 (1.4–1.9) *	1.5 (1.3–1.8) *
	≥ -2 Z-scores	372 (22.9)	1.0	1.0	509 (32.0)	1.0	1.0
**Weight-for-height**	<-2 Z-scores	62 (27.1)	0.9 (0.7–1.3)	NA	37 (29.6)	0.7 (0.5–1.1)	0.6 (0.4–1.0)
	≥ -2 Z-scores	777 (28.2)	1.0	NA	1114 (37.1)	1.0	1.0
**Wealth status**	Poor	300 (31.4)	1.4 (1.1–1.7)*	1.3 (1.1–1.6)*	420 (39.0)	1.3 (1.1–1.6) *	1.3 (1.1–1.6) *
	Middle	281 (27.7)	1.1 (0.9–1.4)	1.1 (0.8–1.3)	396 (38.1)	1.3 (1.1–1.6) *	1.3 (1.1–1.7) *
	Rich	259 (25.6)	1.0	1.0	333 (33.0)	1.0	1.0
**Education of household head**	Illiterate	378 (28.8)	1.1 (0.8–1.5)	NA	554 (39.4)	1.3 (1.0–1.7) *	1.5 (1.1–2.0) *
	Elementary	203 (28.6)	1.1 (0.8–1.5)	NA	273 (37.0)	1.0 (0.8–1.4)	1.2 (0.8–1.6)*
	Secondary and above	260 (27.0)	1.0	NA	322 (32.8)	1.0	1.0
**Intervention arm**	IRS + LLINs	199 (26.8)	1.0	NA	310 (38.1)	1.0	NA
	LLINs alone	220 (28.6)	1.1 (0.9–1.4)	NA	282 (35.0)	0.9 (0.7–1.1)	NA
	IRS alone	199 (29.1)	1.2 (0.9–1.5)	NA	272 (38.5)	1.0 (0.8–1.3)	NA
	Routine	223 (28.3)	1.1 (0.8–1.4)	NA	287 (35.8)	0.9 (0.7–1.1)	NA

NA: Not Applicable because p >0.25

OR: Odds Ratio

*: Significant at P<0.05

†: bivariate analysis

£: Multivariate analysis

N: Number

### Assessment of anaemia using a dynamic cohort

In the dynamic cohort, 2141 non-anaemic children were enrolled in December 2014, and 1266 children newly joined the study, increasing the cohort to a total of 3407 children. In December 2015, after one year of follow-up we observed 865 new anaemia cases. The overall anaemia incidence rate was 30; (95% CI, 28–32) cases per 100 children years of observation. As shown in Table 4, children aged less than 36 months (HR = 3.2), children having malaria (HR = 10.4), and children living in poor families (HR = 1.2) had higher risks of anaemia. However, we did not observe significant differences in risk of anaemia among the trial arms.

**Table 4:** Incidence rate and hazard ratio of anaemia among dynamic cohort of children in Adami Tullu district in south-central Ethiopia, 2014 to 2015.

Variables		CYO	Anaemia cases	IR/100 CYO (95% CI)	Unadjusted † HR (95% CI)	Adjusted £ HR (95% CI)
**Sex**	Boys	1473	442	30 (28–33)	1.1 (0.9–1.2)	NA
	Girls	1440	423	29 (27–32)	1.0	NA
**Age group in months**	6–35	1084	561	52 (48–56)	2.9 (2.6–3.4) *	3.2 (2.8–3.7)*
	36–59	1405	303	22 (19–24)	1.0	1.0
**Malaria status¶**	Positive	46	19	41 (26–64)	8.5 (3.1–23.4)*	10.4 (3.8–28.8) *
	Negative	2866	846	30 (28–32)	1.0	1.0
**Height for age**	> = -2 Z-score	1124	406	36 (33–40)	1.0	1.0
	<-2 Z-score	1106	458	41 (38–45)	1.1 (1.0–1.3) *	1.2 (1.04–1.4)*
**Weight for height**	> = -2 Z-score	1755	314	18 (16–20)	1.0	NA
	<-2 Z-score	134	22	16 (11–25)	0.9 (0.6–1.5)	NA
**Wealth status**	Poor	945	312	33 (30–37)	1.3 (1.1–1.5) *	1.2 (1.04–1.4)*
	Middle	984	300	30 (27–34)	1.2 (1.0–1.4)	1.2 (1.0–1.4)
	Rich	980	251	26 (23–29)	1.0	1.0
**Education of household head**	Illiterate	1707	551	32 (30–35)	1.2 (0.9–1.5)	1.2 (0.9–1.5)
	Elementary	868	226	26 (23–33)	1.0 (0.8–1.2)	0.9 (0.7–1.2)
	Secondary and above	322	86	27 (22–33)	1.0	1.0
**Intervention arms**	LLINs + IRS	765	240	31 (28–36)	1.1 (0.9–1.3)	NA
	LLINs alone	722	207	29 (25–33)	1.0 (0.8–1.2	NA
	IRS alone	661	201	30 (26–35)	1.0 (0.8–1.3)	NA
	Routine	764	217	28 (25–32)	1.0	NA

HR: Hazard Ratio IR: Incidence Rate CYO: Children Years of Observation NA: Not Applicable, because P>0.25 in bivariate analysis

†: bivariate analysis

£: Multivariate analysis

¶: malaria was entered as time varying covariate

### Anaemia among children completed the one year follow-up study (closed cohort)

Out of 2984 children included in the 2014 survey, 1851 (62%) were also examined in 2015. The main reasons for lost to follow-up were exclusion of children older than 59 months (64.1%) and out-migration to other districts (19.5%). Among the 221children who migrated to other districts, 100 (45.2%) were from poor, 72 (32.6%) were from the middle and 49 (22.2%) were from more wealthy families. The overall prevalence of anaemia among children who migrated out was 45.7% (101 children), and this prevalence was higher than the prevalence of anaemia among children who took part in both surveys, 32.9% (610 children) (P<0.001).

Two hundred eighty (46.3%) of children with anaemia in 2014 were also having the same condition in 2015. Of 1851 children participating in both surveys, 1244 children were non-anaemic in December 2014. This cohort of non-anaemic children (n = 1244) was followed for one year (making 1223 children years of observation). We observed 336 new anaemia cases after one year of follow-up in December 2015. The overall incidence of anaemia was 28; (95% CI, 25–31) cases per 100 children years of observation.

As shown in Table 5, anaemia incidence was higher among children less than 36 months (HR = 1.5), having malaria (HR = 4.0), were stunted (HR = 1.2) and among children living in poor families (HR = 1.4). We did not observe statistical significant difference in anaemia among malaria intervention arms.

**Table 5:** Incidence and hazard ratio of anaemia among closed cohort of children in Adami Tullu district, south-central Ethiopia, 2014 to 2015.

Variables		CYO	Anaemia cases	IR/100 CYO (95% CI)	Unadjusted † HR (95% CI)	Adjusted £ HR (95% CI)
**Sex**	Boys	612	176	29 (25–34)	1.1 (0.9–1.4)	NA
	Girls	611	160	26 (22–31)	1.0	NA
**Age group in months**	6–35	415	155	37 (32–44)	1.7 (1.3–2.1) *	1.5 (1.2–1.9)*
	36–59	808	181	22 (19–26)	1.0	1.0
**Malaria status¶**	Positive	26	9	34(18–65)	3.02 (0.9–10.5)	4.0 (1.1–14.3)*
	Negative	1197	327	27 (25–31)	1.0	1.0
**Height for age**	> = -2 Z-score	610	143	23 (20–28)	1.0	1.0
	<-2 Z-score	613	193	32 (27–36)	1.3 (1.1–1.7) *	1.3 (1.05–1.63)*
**Weight for height**	> = -2 Z-score	1178	324	28 (25–31)	1.0	NA
	<-2 Z-score	45	12	27 (15–47)	0.9 (0.5–1.7)	NA
**Wealth Status**	Poor	367	110	30 (25–36)	1.4 (1.1–1.8) *	1.4 (1.05–1.8)*
	Middle	420	135	32 (27–38)	1.5 (1.1–1.9) *	1.5 (1.2–2.0)*
	Rich	413	91	22 (18–27)	1.0	1.0
**Education of household head**	Illiterate	688	197	29 (25–33)	1.1 (0.8–1.6)	1.1 (0.8–1.6)
	Elementary	402	105	26 (22–32)	1.0 (0.7–1.5)	1.0 (0.7–1.5)
	Secondary and above	133	34	25 (18–36)	1.0	1.0
**Intervention arms**	LLINs + IRS	313	98	31 (26–38)	1.1 (0.9–1.5)	NA
	LLINs alone	311	76	24 (20–31)	0.9 (0.7–1.2	NA
	IRS alone	272	71	26 (21–33)	0.9 (0.7–1.3)	NA
	Routine	327	91	28 (23–34)	1.0	NA

HR: Hazard Ratio IR: Incidence Rate CYO: Children Years of Observation NA: Not Applicable, because P>0.25 in bivariate analysis

**†:** bivariate analysis

£: Multivariate analysis

¶: malaria was entered as time varying covariate

Overall, 63 malaria cases (52% *plasmodium falciparum*, 37% *plasmodium vivax*, and 11% mixed infection) were identified during one year follow-up among children participating in both surveys. Among the 63 malaria cases, only 25 (40%) were registered during the major malaria transmission (September to December, 2015). The incidence rate of malaria was 6.7 cases per 10,000 person weeks of observation (95% CI, 5.2–8.5).

## Discussion

This study was a part of a trial to prevent malaria; and showed that low age, stunting, malaria and poverty were the main predictors of anaemia. Although we had expected a reduction of anaemia due to the effect of the malaria prevention trial, we observed an increase in anaemia prevalence among children aged 6 to 59 months from 28% in 2014 to 37% in 2015. Our study took place during a period when there was 60% less rain. The population experienced severe food shortage followed by an increase in the prevalence of stunting. The prevalence of anaemia and stunting were particularly high among children in the poor families, or among families who moved out of the area, probably because of lack of food. Many (36.5%) of the children who were anaemic in 2014 were also anaemic a year later. This could indicate that children remained chronically anaemic, or that they suffered from recurrent anaemia.

In Ethiopia, most of anaemia studies were done as cross-sectional studies [7, 22]. However, we followed the population for one year to measure changes in anaemia prevalence and to assess the incidence and risk factors for anaemia. As our study population was randomly selected from the base population around Lake Zeway in the Rift Valley, we believe the population is representative of rural population living in similar ecological settings. Thus, our findings can be generalized to similar areas in Ethiopia.

The prevalence of anaemia in our 2015 survey (36.8%) was similar to studies from northern Ethiopia (37%) [22], and western Ethiopia (32%) [7]. In agreement with other studies [16, 38], anaemia was common among all age groups, and the prevalence of anaemia decreased with an increase in age. This could be due to increased requirement of iron during periods of rapid child growth. Inadequate intake of iron rich foods and repeated infections in the low age group could result in an increased risk of anaemia in this age group [39].

Malaria parasites invade and destroy red blood cells, and increase the risk of anaemia in children infected with malaria parasite [8]. In this study, the risk of anaemia was higher among children having malaria compared to children without malaria infection, and this supported previous studies [6, 14, 40].

Children in poor families were more at risk of anaemia, as has been reported by others [38, 41]. The poor the families are the less likely to afford adequate and diversified foods, and they are also less likely to seek early treatment for anaemia [42].The observed increased risk of anaemia among children with stunting was consistent with previous research findings [12, 21].This can be related to deficiency of protein energy that could result in impaired immune function with repeated infection that depletes iron stores [43]. Micronutrient deficiencies, including iron, also occur when there is under-nutrition due to food shortage and food lacks diversity [42].

We expected a decrease in prevalence of anaemia among children related to an ongoing malaria prevention trial. However, an increase in the prevalence of anaemia between the two surveys, and high anaemia incidence (30 per 100 person years of observation) could be due to drought and food shortages [26]. In addition, the shortage of water related to decrease in rainfall could also result in poor hygiene and increase occurrence of intestinal parasitic infestation that could contribute to an increase in the prevalence of anaemia [44].

We observed no statistically significant difference in risk of anaemia among the trial arms. This could be related to low malaria incidence. In this study, the observed malaria incidence (6.7 cases per 10,000 children weeks) was lower than our pilot study (8 cases per 10,000 person weeks for the general population, and 11 cases per 10,000 children weeks) as reported elsewhere by Gari et al [28]. The observed low malaria incidence in this study could be due to the short duration and reduced rainfall as a result of the severe drought that affected the area [27].

The present study had some limitations. In the cohort study, children from poor families accounted for 45.2% of all children who left the study area, and the loss to follow-up was most likely related to drought, and to food shortage triggered by El Nino that affected the study area in 2015 and early 2016 [26]. The high prevalence of anaemia among children who left the area (46%) compared to those who completed the two surveys (33%) could have introduced bias in the estimation of the prevalence and incidence of anaemia. The other limitations were: We included children in the age group of 6 to 59 months old, and those older than 59 months were excluded at the beginning of the study. And by doing so, we unfortunately remained with a smaller sample size. Moreover, the low malaria incidence (smaller sample size than expected) could have under-powered the study to detect the effect difference. The causes of anaemia are multi-factorial, and our study could have benefitted from a more thorough laboratory analysis of the causes of anaemia including stool examination for intestinal helminthes. Unfortunately, we only measured some of the main risk factors of anaemia, such as nutritional status assessed using anthropometry, diagnosed malaria status, and socioeconomic variables. Unlike other studies [6, 15, 21], we did not assess the iron status by measuring serum ferritin, and we did not assess the intestinal parasitic load, which could be potential causes of anaemia. Therefore, future anaemia and malaria prevention studies in drought prone areas should include a more thorough assessment of potential causes of anaemia.

In this study, under-estimation of anaemia incidence rate could resulted from: A year apart anaemia survey may not have captured all the anaemia cases in the year time, particularly anaemia cases that occurred and recovered between the two surveys. In addition, the onset of anaemia was uncertain related to the less frequent, once a year anaemia survey we conducted. Therefore, the time to anaemia was defined as the time from malaria diagnose to anaemia diagnosis. However, the time at onset of anaemia could have occurred earlier than the time at anaemia diagnosis, and the use of time to anaemia diagnosis as end point could result in under-estimation of anaemia incidence.

## Conclusion

In conclusion, young age, stunting, malaria and poverty were the main predictors of anaemia. An unexpected increase in the prevalence of anaemia was observed over a year period, and anaemia prevalence was particularly high among people who moved from the area due to lack of food. We did not observe statistical significant difference in risk of anaemia among malaria intervention arms. Therefore, conducting trials in settings prone to drought and food shortage is a serious challenge. Further study could be needed to measure serum ferritin value to establish the proportion of iron deficiency anaemia among the children.
